# Turn TRAIL Into Better Anticancer Therapeutic Through TRAIL Fusion Proteins

**DOI:** 10.1002/cam4.70517

**Published:** 2024-12-30

**Authors:** Yan Wang, Xin Qian, Yubo Wang, Caiyuan Yu, Li Feng, Xiaoyan Zheng, Yaya Wang, Qiuhong Gong

**Affiliations:** ^1^ College of Agroforestry and Medicine The Open University of China Beijing China; ^2^ Endocrinology Centre Fuwai Hospital, Chinese Academy of Medical Sciences and Peking Union Medical College Beijing China; ^3^ Department of Pharmacy Beijing Ditan Hospital Capital Medical University Beijing China; ^4^ State Key Laboratory of Quality Research in Chinese Medicines Macau Institute for Applied Research in Medicine and Health, Macau University of Science and Technology Macau China

**Keywords:** apoptosis, cancer, fusion protein, resistance, TRAIL

## Abstract

**Background:**

TNF‐related apoptosis‐inducing ligand (TRAIL) belongs to the tumor necrosis factor superfamily. TRAIL selectively induces apoptosis in tumor cells while sparing normal cells, which makes it an attractive candidate for cancer therapy. Recombinant soluble TRAIL and agonistic antibodies against TRAIL receptors have demonstrated safety and tolerability in clinical trials. However, they have failed to exhibit expected clinical efficacy. Consequently, extensive research has focused on optimizing TRAIL‐based therapies, with one of the most common approaches being the construction of TRAIL fusion proteins.

**Methods:**

An extensive literature search was conducted to identify studies published over the past three decades related to TRAIL fusion proteins. These various TRAIL fusion strategies were categorized based on their effects achieved.

**Results:**

The main fusion strategies for TRAIL include: 1. Construction of stable TRAIL trimers; 2. Enhancing the polymerization capacity of soluble TRAIL; 3. Increasing the accumulation of TRAIL at tumor sites by fusing with antibody fragments or peptides; 4. Decorating immune cells with TRAIL; 5. Prolonging the half‐life of TRAIL in vivo; 6. Sensitizing cancer cells to overcome resistance to TRAIL treatment.

**Conclusion:**

This work focuses on the progress in recombinant TRAIL fusion proteins and aims to provide more rational and effective fusion strategies to enhance the efficacy of recombinant soluble TRAIL, facilitating its translation from bench to bedside as an effective anti‐cancer therapeutic.

## Graphical abstract

This review comprehensively covers the TRAIL fusion proteins that have been reported in recent years. It also summarizes six commonly employed strategies used in the construction of TRAIL fusion proteins.

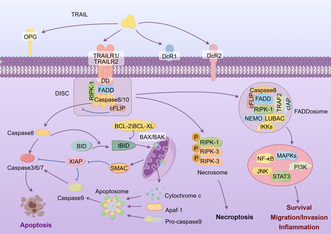

**Abbreviations**
ABD: albumin‐binding domain
ADCC: antibody‐dependent cell‐mediated cytotoxicity
Apaf‐1: apoptotic protease activating factor 1
BAK: BCL‐2 homologous antagonist killer
BAX: B‐cell lymphoma 2‐associated X
BCL‐2: B‐cell lymphoma 2
BID: BH3‐interacting domain death agonist
c‐FLIP: cellular FLICE‐inhibitory protein
DcR1: decoy receptor 1
DcR2: decoy receptor 2
DISC: death‐inducing signaling complex
DR4: death receptor 4
DR5: death receptor 5
EGFR: epidermal growth factor receptor
EHD2: heavy‐chain domain 2
ELPs: elastin‐like polypeptides
ERK: extracellular signal‐regulated kinase
FADD: Fas‐associated protein with death domain
FeSOD: iron superoxide dismutase
Fn14: fibroblast growth factor‐inducible 14
HER2: human epidermal growth factor receptor 2
IAP: inhibitor of apoptosis
IgBDs: immunoglobulin‐binding domains
IgE: immunoglobulin E
JNK: c‐Jun N‐terminal kinase
LUBAC: linear ubiquitin chain assembly complex
LZ: leucine zipper
MAPK: mitogen‐activated protein kinase
NEMO: NF‐κB essential modulator
NF‐kB: nuclear factor kappa‐light‐chain‐enhancer of activated B‐cells
NK cells: natural killer cells
OPG: osteoprotegrin
PI3K: phosphatidylinositide 3‐kinases
RIPK1: receptor‐interacting serine/threonine protein kinase 1
RIPK3: receptor‐interacting serine/threonine protein kinase 3
scFv: single‐chain variable fragments
scTRAIL: single‐chain trimeric TRAIL
SMAC: second mitochondria‐derived activator of caspase
STAT3: signal transducer and activator of transcription 3
tBID: truncated BH3‐interacting domain death agonist
TNC: tenascin‐C
TNFRSF: tumor necrosis factor receptor superfamily
TNFSF: tumor necrosis factor superfamily
TRAF2: TNF receptor‐associated factor 2
TRAIL: tumor necrosis factor‐related apoptosis‐inducing ligand
TWEAK: tumor necrosis factor‐like weak inducer of apoptosis
VEGFR2: vascular endothelial growth factor receptor 2
XIAP: X‐linked inhibitor of apoptosis protein

## 1 Introduction

Tumor necrosis factor‐related apoptosis‐inducing ligand (TRAIL), also known as Apo‐2 ligand (Apo2L), belongs to the tumor necrosis factor superfamily (TNFSF) [1]. TRAIL has demonstrated the ability to induce apoptosis in cancer cells while causing little damage to normal cells, making it a promising candidate for cancer therapy. Since its discovery nearly three decades ago, recombinant soluble TRAIL (sTRAIL) and monoclonal antibodies targeting agonistic TRAIL receptors have been developed and evaluated in both preclinical and clinical studies. Although these drugs have shown good tolerability in patients during clinical trials, they have failed to elicit compelling objective responses either as monotherapy or in combination with other therapeutic agents [2].

Nevertheless, there is still considerable interest in developing TRAIL‐based therapeutics as cancer‐selective drugs by enhancing the pharmacokinetic and/or pharmacodynamic properties of TRAIL. One significant approach is the construction of genetically engineered fusion proteins that combine recombinant sTRAIL with other functional proteins or polypeptides. In recent years, many elegantly designed recombinant sTRAIL fusion proteins have been reported, with an increasingly wide range of fusion partners. Some of these have demonstrated superior anticancer activity, and several have entered clinical trials. This review focused on the progress in recombinant TRAIL fusion proteins, including updates on their clinical trials in order to provide more rational and effective fusion strategies to enhance the efficacy of recombinant sTRAIL and facilitate its development for cancer therapy.

## 2 TRAIL Signaling

As an immunocyte cytokine, TRAIL is expressed as a homotrimeric type II transmembrane protein on the surfaces of various immunocytes including natural killer (NK) cells [3], activated T cells, monocytes, dendritic cells, and macrophages. It plays a role in regulating immune surveillance [1]. The trimeric structure of TRAIL relies on the coordination of three Cys‐230 residues from each monomer with one Zn^2+^ ion at its trimeric core, and the Zn^2+^ ion is also essential for maintaining stability and biological activity for the trimeric TRAIL [4]. Similar to other members of the TNFSF family, the extracellular domain of membrane‐bound TRAIL can be cleaved from the cell membrane to generate sTRAIL, which is also a bioactive homotrimer. However, it has been demonstrated that membrane‐bound TRAIL possesses much stronger cancer cell killing activity than the soluble version [5].

TRAIL interacts with five receptors: TRAIL‐R1/death receptor (DR) 4, TRAIL‐R2/DR5, TRAIL‐R3/decoy receptor (DcR) 1, TRAIL‐R4/DcR2, and osteoprotegrin (OPG) [6]. Both DR4 and DR5 serve as agonistic receptors, with their extracellular domain containing a preligand assembly domain (PLAD) that promotes the oligomerization of the DR4 and DR5 trimers to enhance binding with TRAIL [7]. The intracellular domains of DR4 and DR5 contain the full‐length intracellular death domain (DD), a conserved motif crucial for cell death induction [8]. There are two decoy TRAIL receptors: DcR1 lacks a cytosolic region while DcR2 has a truncated, non‐functional cytoplasmic DD. OPG is a soluble receptor without an intracellular domain. These three receptors cannot initiate cell death signaling, but they act as decoys competitively inhibiting the binding of TRAIL to DR4 and DR5 [9].

**FIGURE 1:**
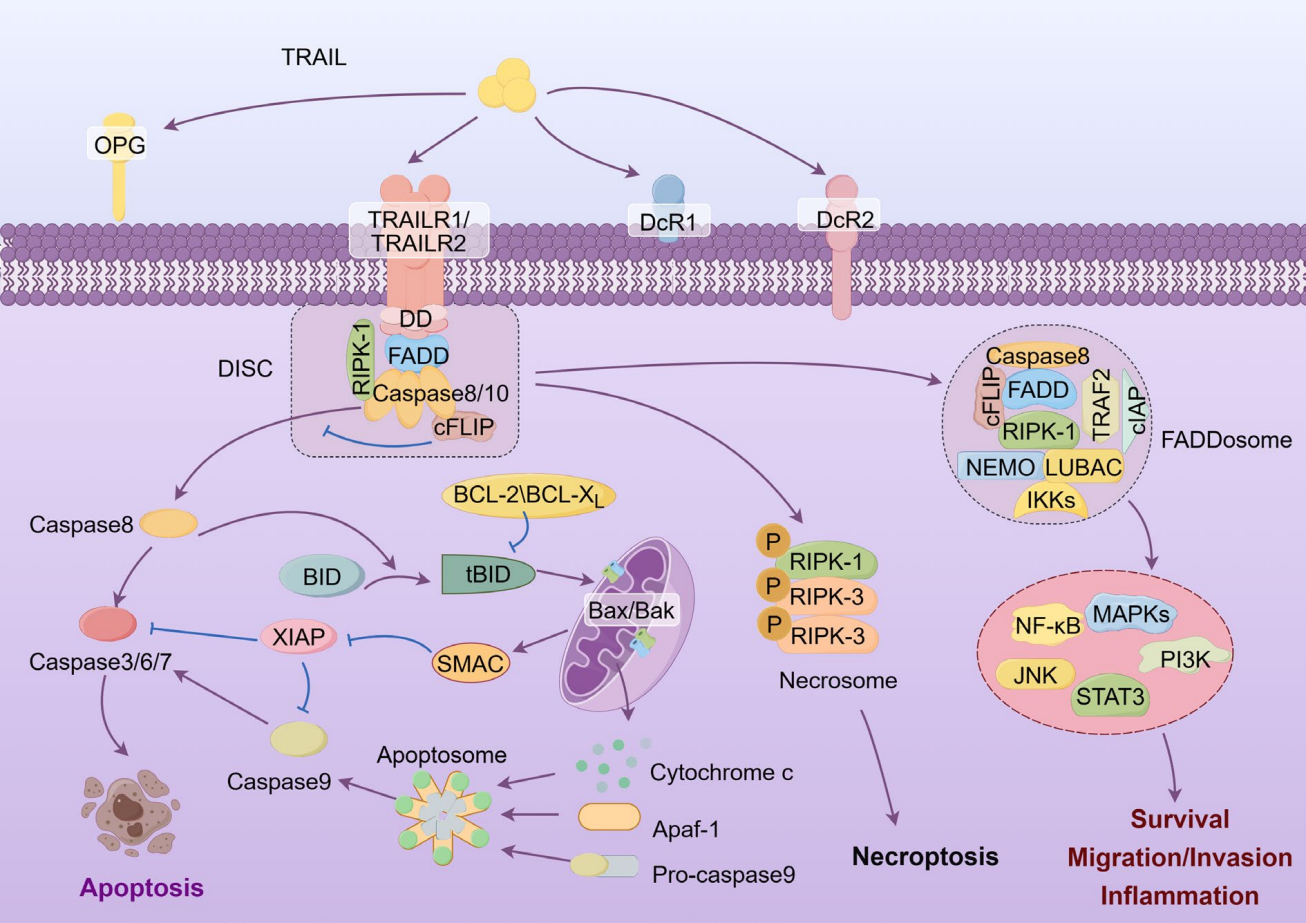
The apoptotic and non‐apoptotic pathways of TRAIL.

Ligation of TRAIL trimers to DR4 and DR5 initiates receptors trimerization, then leading to the formation of higher‐order complexes [10]. The crosslinked receptors lead to cross‐linking of their intracellular DDs. Then, their DDs recruit the intracellular adaptor Fas‐associated protein with death domain (FADD), which further recruits initiator caspases: procaspase‐8, and/or procaspase‐10, to form the death‐inducing signaling complex (DISC) [11]. Procaspase‐8/10 within the DISC is then activated via proteolytic cleavage, and following activates downstream effector proteins caspase‐3, ‐6, and ‐7. Activated effector caspases then cleave vital cellular proteins, ultimately causing apoptosis. This pathway is referred to as the “extrinsic apoptosis pathway” [12]. In some cells, TRAIL triggers the intrinsic apoptotic pathway by the caspase‐8‐mediated cleavage of BH3‐interacting domain death agonist (BID) to generate truncated BH3‐interacting domain death agonist (tBID). tBID subsequently binds and activates the proapoptotic proteins B‐cell lymphoma 2 (BCL‐2)‐associated X (BAX) and BCL‐2 homologous antagonist killer (BAK), resulting in mitochondrial outer membrane permeabilization, and the subsequent release of cytochrome c and the second mitochondria‐derived activator of caspase (SMAC) into the cytoplasm [13]. Cytochrome c then complexes with apoptotic protease activating factor 1 (Apaf‐1) and procaspase‐9 to form the apoptosome, which activates caspase‐9 followed by the hydrolysis of caspase‐3, ‐6, and ‐7, ultimately resulting in cellular apoptosis [14] (Figure 1). The extent to which DR4 and DR5 induce apoptosis in cancer cells via extrinsic or intrinsic pathways remains incompletely characterized and appears to differ depending on the cell type.

In addition to its role in apoptosis induction, TRAIL binding with DR4 and DR5 may also elicit noncanonical signaling pathways. Under certain conditions, such as caspase‐8 deficiency or inactivation [15], receptor‐interacting serine/threonine protein kinase 1 (RIPK1) is recruited to the TRAIL DRs, which then recruits and phosphorylates RIPK3, leading to the formation of the necrosome and the induction of necroptosis [16] (Figure 1).

TRAIL can also trigger inflammatory and cell survival pathways, although the signaling mechanisms are not fully understood. Ligation of TRAIL receptors leads to the formation of DISC (complex I), which presumably dissociates from the receptors and assembles into a secondary cytoplasmic complex known as the FADDosome (complex II) [17, 18]. The FADDosome consists of caspase‐8, FADD, and RIPK1, along with cellular FLICE‐inhibitory protein (c‐FLIP) [17], caspase‐10, TNF receptor‐associated factor 2 (TRAF2), inhibitors of apoptosis (IAPs), NF‐κB essential modulator (NEMO) [19], the linear ubiquitin chain assembly complex (LUBAC) [20], and other regulatory molecules. This complex then leads to inflammation or cell survival via pathways such as nuclear factor κB (NF‐κB), mitogen‐activated protein kinase (MAPK) including p38, phosphoinositide 3‐kinases/protein kinase B (PI3K/Akt), extracellular signal‐regulated kinase (ERK), c‐Jun N‐terminal kinase (JNK), and signal transducer and activator of transcription 3 (STAT3) [21] (Figure 1). Furthermore, emerging evidence suggests that DcR2 may induce noncanonical TRAIL signaling through its truncated intracellular domain [22].

## 3 Dilemma of TRAIL Research

Given TRAIL's ability to selectively induce apoptosis in tumor cells, recombinant human TRAIL and TRAIL‐receptor agonistic antibodies have been developed as anticancer therapeutics. These agents, classified as first‐generation TRAIL receptor agonists (TRAs), have demonstrated promising preclinical antitumor activity by inducing apoptosis in various tumor cell types [23]. Clinical trials have shown that these agents are well tolerated by patients, even at high doses. However, they seem to elicit a limited therapeutic efficacy.

Agonistic antibody to DR4, such as Mapatumumab [24, 25, 26, 27, 28], and agonistic antibody to DR5, including Conatumumab [29, 30], Tigatuzumab [31, 32, 33], Drozitumab [34], Lexatumumab [35, 36], and LBY135 [37], have been evaluated in Phase 1 and/or 2 clinical trials as monotherapy or in combination for the treatment of various cancers. These antibodies demonstrated acceptable safety and tolerability, but their efficacy was generally underwhelming, with only negligible benefits, if any, reported.

Dulanermin, the first recombinant soluble human TRAIL (aa114‐281) [38], has completed clinical trials from Phase 1 to Phase 3 in patients of non‐small cell lung cancer [39, 40, 41], B‐cell lymphomas [42], colorectal cancer [43], or non‐Hodgkin's lymphoma [44], either as monotherapy or in combination with other anticancer therapies. These trials demonstrated that dulanermin was safe and well tolerated. However, the clinical efficacy was rather limited [2, 45]. Combining TRAIL with paclitaxel, carboplatin (PC), and bevacizumab (PCB) did not improve the patients' clinical outcomes [40] or elicit rather limited therapeutic efficacy [41]. Another recombinant TRAIL variant, circularly permuted TRAIL (CPT, Aponermin), has entered Phase 1, 2, and 3 studies [46, 47, 48, 49]. CPT in combination with thalidomide and dexamethasone was well tolerated and showed significant improvements in progression‐free survival (PFS), overall survival (OS), and overall response rate (ORR) in patients with relapsed or refractory multiple myeloma in the Phase 3 trial. However, the investigators found that this benefit was still limited compared to that of other new drugs [49].

The challenges encountered by TRAIL and TRAs in transitioning from bench to bedside are primarily attributed to three main factors: pharmacokinetics, pharmacodynamics, and resistance. Recombinant sTRAIL has a short serum half‐life (0.56–1.02 h in Phase 1 trials in patients [38]), which leads to rapid systemic clearance via renal excretion [50]. Consequently, repeated administration is necessary for maintaining therapeutic levels in circulation. Furthermore, membrane‐bound TRAIL is more potent (100‐ to 1000‐fold) as an inducer of cell death compared to sTRAIL due to its ability to oligomerize or cluster DR4 and DR5, facilitating more efficient downstream signal transduction [51, 52]. In contrast, recombinant sTRAIL or agonistic TRAIL receptor antibodies struggle to anchor to cell membranes, resulting in inefficient clustering of TRAIL receptors and lower effective apoptotic signal transduction than membrane‐bound TRAIL [51]. Additionally, many tumor cells exhibit inherent or acquired resistance toward TRAIL or agonistic TRAIL receptor antibodies through various mechanisms.

Therefore, to enhance the clinical efficacy of TRAIL, efforts should be focused on these three areas.

## 4 Construction of Recombinant TRAIL Fusion Proteins

To address the limitations of TRAIL‐based therapy, numerous strategies have been investigated to enhance the antitumor efficacy of TRAIL, including the engineering of TRAIL fusion proteins, combination with chemotherapeutic drugs, developing TRAIL sensitizers, TRAIL gene therapy, and targeted delivery of TRAIL by nanocarriers or host cells (reviewed in [2]). Among these strategies, the construction of recombinant TRAIL fusion proteins stands out as an efficacious and fundamental approach, which is to fuse the DNA of sTRAIL with DNA encoding one or more functional peptides/proteins with genetic engineering methods. Then the resulting recombinant construct is transformed into prokaryotic cells (e.g., Escherichia coli) or eukaryotic cells (e.g., CHO cells) to express the fusion proteins.

### 4.1 Construction of Stable TRAIL Trimers

As mentioned above, the structure of homotrimer is prerequisite for the biological activity of TRAIL. However, recombinant soluble trimeric TRAIL is prone to instability, leading to dissociation into monomers and subsequent formation of disulfide‐linked dimers, which exhibit significantly reduced apoptotic activity compared to trimers [53]. Therefore, the primary focus in the development of TRAIL fusions is on achieving stable trimer formation. Covalently linking three soluble recombinant TRAIL monomers into a single polypeptide promotes trimerization, resulting in improved stability and cytotoxicity comparable to native TRAIL. This fused single‐chain trimeric TRAIL (scTRAIL) often serves as a tool or module for constructing more intricate TRAIL fusion proteins [54].

Another effective method to obtain trimeric TRAIL is to fuse TRAIL with domains capable of spontaneous trimerization, such as leucine zipper (LZ) [55], tenascin‐C (TNC) [56], the C‐terminal fiber shaft repeat of human adenovirus type 5 (Ad5) fiber protein [57], the triple helix domain of human collagen XVIII [58], the c‐propeptide of α1(I) collagen [59], and the trimer coiled helix domain of human lung surfactant‐associated protein D [60]. Overall, trimeric TRAIL fusion proteins exhibit activity similar to or slightly better than that of native TRAIL in *in vitro* assays of tumor cell activity. However, the trimeric form of the fusion proteins is more stable and binds zinc ions more tightly [59], resulting in stronger antitumor activity *in vivo*. The increased molecular weight of fusion TRAILs also results in an extended half‐life.

Among these trimeric fused TRAIL variants, SCB‐313, which is a fusion of TRAIL with the c‐propeptide of α1(I) collagen, has already completed Phase 1 clinical trials in China and Australia (NCT04051112, NCT04123886, NCT03443674, and NCT03869697) for the treatment of malignant peritoneal tumors and malignant pleural effusion. Results from NCT04051112 and NCT03443674 have demonstrated that SCB‐313 exhibits acceptable safety profiles and a reduction in ascites flow rate at therapeutic doses [61]. However, there have been no reports of Phase 2 clinical trials of SCB‐313 to date.

### 4.2 Promoting the Formation of TRAIL Polymers

Similar to other members of the tumor necrosis factor receptor superfamily (TNFRSF) [10, 62], forming higher order assemblies of DR4 or DR5 based on the trimeric structure has been shown to more effectively activate the receptors and induce significantly amplified apoptosis. This is supported by observations that secondary cross‐linking of FLAG‐tagged TRAIL, achieved through the addition of anti‐FLAG antibodies, exhibits markedly enhanced tumor cell apoptosis compared to native TRAIL [63, 64]. Consequently, it is plausible that the insufficient cross‐linking of sTRAIL may be a contributing factor to its limited antitumor efficacy in clinical trials.

Therefore, constructing polymeric TRAIL becomes an attractive option for enhancing therapeutic efficacy of TRAIL to promote higher order oligomerization of TRAIL receptors (Table 1). The fusion of TRAIL with a hexametric domain of the isoleucine zipper motif, known as ILz(6):TRAIL, has been observed to form hexamers and higher order polymers, resulting in enhanced apoptosis‐inducing activity compared to the trimeric form of TRAIL [65]. Immunoglobulins are natural covalently linked homodimer that can serve as scaffolds for the construction of polymeric TRAIL. For instance, fusion of the heavy‐chain domain 2 (EHD2) of immunoglobulin E (IgE) with scTRAIL resulted in a hexavalent TRAIL with enhanced antitumor activity [67]. Similarly, fusion of scTRAIL to either the light chain, heavy chain, or both chains of an anti‐EGFR IgG antibody produced hexavalent or dodecavalent IgG‐scTRAIL molecules, all of which exhibited increased bioactivity. Notably, dodecavalent scTRAIL demonstrated superior bioactivity compared to the hexavalent IgG‐scTRAIL [68]. Moreover, fusion of the Fc‐part of a human IgG1‐mutein with two scTRAIL‐receptor‐binding domain polypeptides also created a hexavalent scTRAIL‐RBD dimer known as APG350 [77] (with an optimized version named ABBV‐621 [69]), with increased antitumor activity *in vitro* and *in vivo*. Encouragingly, ABBV‐621 has advanced to clinical trials. A completed Phase 1 trial (NCT03082209) evaluated its safety, efficacy, and pharmacokinetic profile in patients with previously treated solid tumors or hematologic malignancies. This study demonstrated the acceptable safety and preliminary antitumor effects of ABBV‐621 [78]. Another Phase 1b clinical trial (NCT04570631) is ongoing in patients with relapsed or refractory multiple myeloma that aimed to determine the efficacy of ABBV‐621 in combination with bortezomib and dexamethasone [79].

**TABLE 1:** Polymeric TRAIL fusion proteins.

Fusion partner	Name	Polymeric state	Reference
Isoleucine zipper motif	ILz(6):TRAIL	Hexamers and higher order polymers	[65]
α‐Helical coils from MBD2 and p66α proteins	HexTR	Hexamer	[66]
Heavy‐chain domain 2 of IgE	scFv‐EHD2‐scTRAIL	Hexamer	[67]
Light chain of anti‐EGFR IgG	LC‐scTRAIL	Hexamer	[68]
Heavy chain of anti‐EGFR IgG	HC‐scTRAIL	Hexamer	[68]
Light and heavy chains of anti‐EGFR IgG	LC/HC‐scTRAIL	Dodecamer	[68]
Fc‐part of a human IgG1‐mutein	APG350 or ABBV‐621	Hexamer	[69]
Multivalent protein scaffold (MV) by adding tailpiece of IgM to IgG Fc	MV‐TRAIL	Polymers	[70]
TGF3L peptide	TGF3L‐TRAIL	Polymers	[71]
NCTR_25_ peptide	NCTR_25_‐TRAIL	Polymers	[72]
NCTR_25_‐TGF3L	NCTR_25_‐TGF3L‐TRAIL	Polymers	[72]
Elastin‐like polypeptides	RGD‐TRAIL‐ELP	Nanoparticles	[73]
Ferritin	TRAIL‐ATNC^IL4rP^	Active trimer nanocage	[74]
SnoopTagJr/SnoopDogTag	SnHexaTR	Trimer or hexamer when adding SnoopLigase	[75]
Fn14	Fn14.TRAIL	Adding TWEAK to form oligomer	[76]

In addition to forming oligomers, some TRAIL fusion proteins can form high‐order assemblies, even reaching nanoscale. Elastin‐like polypeptides (ELPs) are temperature‐sensitive biopolymers derived from human tropoelastin. When fused with TRAIL, the resulting fusion protein can spontaneously assemble into nanoparticles at 37°C with improved apoptosis‐inducing bioactivity [73]. Additionally, TRAIL fused with the triple helical domain of pulmonary surfactant‐associated protein D and ferritin, known for its ability to form a cage‐like supramolecular assembly, produced TRAIL‐ATNC, which formed nanoparticles with prolonged serum half‐life and enhanced tumor targeting [74]. We have reported a series of self‐assembled stable TRAIL polymers, including TGF3L‐TRAIL [71], NCTR_25_‐TRAIL, and NCTR_25_‐TGF3L‐TRAIL [72], and their hydrodynamic radius measured by DLS was 30–40 nm on average. These polymers demonstrated significantly enhanced tumor cell‐specific cytotoxicity *in vitro*, with an LD_50_ four orders of magnitude lower than sTRAIL in Colo205 cells, and also showed improved *in vivo* activity compared to recombinant sTRAIL.

Additionally, the polymerization of TRAIL can be achieved by adding enzymes or ligand. The fusion of TRAIL with minimal superglue peptide pairs, such as Snoopligase‐catalyzed SnoopTagJr/SnoopDogTag and SpyStapler‐catalyzed SpyTag/SpyBDTag, resulted in superglue‐fusion TRAIL variants capable of spontaneous trimerization. Upon introduction of Snoopligase or SpyStapler, the trimeric superglue‐fusion TRAIL variants were predominantly crosslinked into hexavalent TRAIL variants, named snHexaTR or spHexaTR [75, 80]. Furthermore, distinct from previously reported fusion proteins, the fusion of TRAIL to fibroblast growth factor‐inducible 14 (Fn14)—a receptor for tumor necrosis factor‐like weak inducer of apoptosis (TWEAK)—is capable of forming trimers. And this fusion protein and undergoes oligomerization in the presence of TWEAK, resulting in increased activity [76].

In addition to enhanced activity, many polymeric TRAIL fusion proteins have shown increased drug accumulation. For example, hexavalent SnHexaTR demonstrated enhanced tumor uptake (over 2 times) than that of trivalent TRAIL [75]; a TRAIL‐active trimer nanocage (TRAIL‐ATNC) showed approximately 1.5 times higher tumor uptake than that of TRAIL [74]. The self‐assembly nanoparticle RGD‐TRAIL‐ELP exhibited 2.5‐fold higher tumor accumulation than that of RGD‐TRAIL [73].

### 4.3 Enhancing the Tumor Targeting of TRAIL

As mentioned above, TRAIL has five corresponding receptors. TRAIL triggers cell apoptosis in cancerous cells due to its ligation to agonistic receptors DR4 and DR5, while sparing normal cells due to the competitively inhibition of DcR1 and DcR2 on normal cells [9]. Nevertheless, the systemic administration of TRAIL was trapped by its sequestration by normal cells that overexpress DcRs, leading to limited uptake by tumor cells. Tumor cells can resist to TRAIL through downregulation or increased internalization of DR4 and DR5, or overexpression of DcRs (DcR1 and DcR2) [81]. In addition, some normal cells, such as hepatocytes, can express DR4 and DR5 [82], leading to concerns about TRAIL‐induced hepatotoxicity. Therefore, enhancing the targeting of TRAIL to tumor sites is essential when optimizing TRAIL.

Molecules overexpressed on the surface of tumor cells provide opportunities for the design of targeted TRAIL fusion proteins. For instance, peptides that target the epidermal growth factor receptor (EGFR) [83, 84, 85] or human epidermal growth factor receptor‐2 (HER2) [85, 86] are frequently used in the design of TRAIL fusion proteins. Additionally, the tumor microenvironment is characterized by vigorous neovascularization, which is vital for tumor proliferation, metastasis, and drug resistance. Targeting neovascularization is therefore another effective cancer treatment strategy [87]. Fusion with targeted fragments gives TRAIL the potential to selectively accumulate in tumor tissues, thereby improving therapeutic outcomes and minimizing harm to normal tissues.

#### 4.3.1 Antibody/Antibody Fragment Fusion With TRAIL

Antibodies are a crucial class of targeted cancer therapeutics known for their high specificity and affinity, long half‐life, and the ability to block receptor–ligand interactions. They also mediate complement‐dependent cytotoxicity (CDC), antibody‐dependent cellular cytotoxicity (ADCC), and antibody‐dependent cellular phagocytosis (ADCP) [88]. Therefore, in addition to serving as targeted therapeutics themselves, antibodies have also been reported to be fused with TRAIL to enhance its targeting and efficacy [68, 89, 90] (Table 2).

**TABLE 2:** TRAIL fusions with antibody/antibody fragments.

Fusion partner	Name	Target	Effect	Reference
Complete antibodies				
CD19 IgG1	CD19‐TRAIL	CD19	Efficiently kills CD19^+^ tumor cells	[89]
Anti‐EGFR huC225 IgG1	LC‐scTRAIL, HC‐scTRAIL, LC/HC‐scTRAIL	EGFR	Efficiently induces EGFR^+^ cell death	[68]
Anti‐MCSP	TRAIL	MCSP	Potently inhibits outgrowth of melanoma	[90]
Antibody fragments				
EGFR targeted nanobody	ENb‐TRAIL	EGFR	Induces apoptosis in cancer cells insensitive to TRAIL	[83]
Diabody of anti‐EGFR	Db‐scTRAIL	EGFR	Shows potent and long‐lasting tumor response	[84]
scFvhu225	scFvhu225‐Fc‐scTRAIL	EGFR	Almost complete tumor remission but early regrowth	[85]
4D5 scFv of anti‐HER2	4D5 scFv‐TRAIL	HER2	Specifically targets to the surface of HER2‐overexpressing tumor cells and inhibits cell growth	[86]
scFv3‐43	scFv3‐43‐Fc‐scTRAIL	HER3	Stable tumor remission	[85]
scFv323/A3hu3	scFv323/A3hu3‐Fc‐scTRAIL	EpCAM	Partial tumor regression and fast regrowth	[85]
Anti‐Kv10.1 nanobody	VHH‐D9‐scTRAIL	Kv10.1	Induces apoptosis in Kv10.1^+^ tumor cells	[91]
scFv:G28‐5	scFv:G28‐TRAIL	CD40	Enhances apoptosis induction and stimulates DC maturation	[92]
scFv:lαhCD70	scFv:lahCD70‐TNC‐TRAIL	CD70	Triggers cell death 10‐ to 100‐fold more efficiently in CD70 positive cells	[93]
scFvCD33	scFvCD33:sTRAIL	CD33	Induces CD33‐targeted apoptosis and shows bystander activity toward CD33^−^ tumor cells	[94]
Anti‐CD38 scFv Diabody	IL2‐αCD38‐αCD38‐scTRAIL	CD38, IL‐2R	Selectively kills CD138^+^ cells	[95]
scFvCD20	scFvCD20‐sTRAIL	CD20	scFvCD20‐sTRAIL secreting mesenchymal stem cells migrates to the tumor site and significantly inhibits the tumor growth	[96]
scFvCD7	scFvCD7:sTRAIL	CD7	Induces potent apoptosis in CD7^+^ malignant T cells and induces bystander apoptosis of CD7^−^ tumor cells	[97]
scFvCD47	Anti‐CD47:TRAIL	CD47	Enhances RTX‐induced B‐NHL cell phagocytosis by granulocytes	[98]
scFvPD‐L1	Anti‐PD‐L1:TRAIL	PD‐L1	Blocks PD‐1/PD‐L1 interaction, enhances T cell activation, and sensitizes cancer cells to TRAIL	[99]
scFvM58	scFvM58‐sTRAIL	MRP‐3	Induces apoptosis toward MRP3^+^ GBM cells	[100]

However, the large molecular weight of full‐length antibodies makes it challenging for constructing fusion proteins. The single‐chain variable fragments (scFvs) of antibodies (26–28 kDa) are more popular in the construction of fusion proteins. ScFvs incorporate the complete antigen‐binding site of an antibody by combining the V_H_ (variable domain of immunoglobulin heavy chain) and V_L_ (variable domain of immunoglobulin light chain) connected with a flexible peptide linker [101], making it a more compact size and comparable antigen‐binding specificity and high affinity to complete antibodies. In recent years, there have been numerous TRAIL fusion proteins constructed using scFvs (Table 2). Alongside scFvs, the diabody (a variant of scFv by reducing the linker between V_H_ and V_L_) has also been employed to construct dimerized targeted TRAIL [84]. Furthermore, nanobodies, another smaller antibody fragments with the V_H_ from camelids antibodies, are also utilized as fusion partners for TRAIL [83, 91].

The fusion of antibodies or antibody fragments with TRAIL led to multifunctional TRAIL fusion proteins, which exhibit enhanced tumor selectivity and reduced off‐target effects. Furthermore, certain antibodies could inhibit cell prosurvival signaling [83, 99] or stimulate dendritic cells maturation [92], thereby synergistically enhancing the antitumor efficacy with TRAIL. Moreover, the high affinity of antibodies allows for temporary anchoring of TRAIL to the cell membrane, mimicking membrane‐bound TRAIL and augmenting its activity. Additionally, the scFv‐TRAIL fusion protein demonstrates a so‐called “bystander effect” by eliminating tumor cells without the specific antigen in close proximity to target cells through a paracrine‐like mechanism [92, 94, 97]. Table 2 presents a summary of TRAIL fusions with their targets and fused antibody/antibody fragments in recent studies.

#### 4.3.2 Peptides Fusion With TRAIL

In addition to antibodies, small peptides/ligands that recognize tumor‐associated antigens/receptors have been utilized as fusion partners for TRAIL to enhance the tumor‐homing capabilities of TRAIL and inhibit tumor growth through alternative molecular pathways. “Z‐domain” or “affibody” is a small recognition molecule derived from domain B of staphylococcal protein A [102], which shows excellent tumor penetration and precisely targets some cancer cells with specific overexpressed molecular signatures [103]. For instance, the fusion of PDGFRβ‐specific affibody Z_PDGFRβ_ with ABD‐TRAIL has been demonstrated to effectively target tumor cells and PDGFRβ‐positive pericytes on tumor microvessels, facilitating the homing of TRAIL to the tumor site [104]. Similarly, the antiangiogenic synthetic peptide SRHTKQRHTALH has been shown to confer the TRAIL variant with the ability to target vascular endothelial growth factor receptor 2 (VEGFR2) [105]. Peptides from RGD and NGR families have also been extensively exploited to enhance the penetration of various compounds by binding to integrins αVβ3 and αVβ5 [106], which are highly expressed in both tumor cells and the tumor vasculature. Table 3 displays the peptides that have been fused with TRAIL in recent studies.

**TABLE 3:** TRAIL fusions with targeting small peptides.

Fusion partner	Name	Target	Effect	Reference
IL‐2	IL2‐TRAIL	IL2 receptor	Induces higher apoptosis in CD25^+^ cells	[107]
IL4 receptor‐binding peptides (IL4rPs)	TRAIL‐ATNC^IL4rP^ nanocages	IL4 receptor	Enhanced *in vivo* stability and antitumor efficacy	[74]
MUC16‐binding domain of mesothelin	Meso64‐TR3	MUC16	More potent than TR3 *in vitro* and *in vivo* in MUC16^+^ ovarian cancer	[108]
CD19L	CD19L‐TRAIL	CD19	Potent *in vivo* antileukemic activity at nontoxic fmol/kg dose levels and slower *in vivo* elimination than TRAIL	[109]
HER2‐specific affibody Z_HER2:342_	TRAIL‐ Affibody	HER2	Increased tumor‐homing ability and antitumor efficiency than those of TRAIL	[110]
PDGFRβ‐specific affibody Z_PDGFRβ_	Z‐ABD‐TRAIL	PDGFRβ	Engagement of PDGFRβ‐expressing pericytes on tumor microvessels and long‐lasting (> 72 h) tumor‐killing ability	[104]
RGR peptide (CRGRRST)	RGR‐TRAIL	Not clear	Improved the tumor uptake and antitumor effects of TRAIL in DR‐overexpressing colorectal cancer cells	[111]
NGR and (or) RGD	RGD‐TRAIL, TRAIL‐NGR, RGD‐TRAIL‐NGR	Integrin ανβ3	Induces more potent apoptosis in cells insensitive to TRAIL	[106]
iRGD (CRGDKGPDC)	DR5‐B‐iRGD	Integrin αvβ3	Penetrates U‐87 tumor spheroids much faster with enhanced antitumor effect than DR5‐B	[112]
Antiangiogenic synthetic peptide SRHTKQRHTALH	SRH‐DR5‐B	VEGFR2	Shows higher efficacy than DR5‐B in 3D tumor spheroids of the HT‐29 and U‐87 cell lines	[105]
RKRKKSR peptides derived from VEGFA	AD‐O51.4	VEGF receptors	Triggers apoptosis in TRAIL‐resistant cancer cells	[113, 114]
Vasostatin	VAS‐TRAIL	Not given	Induces apoptosis in endothelial cells	[115]

### 4.4 Arming Immune Cells With TRAIL

TRAIL, expressed on the surface of various innate and adaptive immune cells [116, 117, 118, 119, 120], plays a pivotal role in tumor immune surveillance [6]. It collaborates with FasL and perforin to exert cytotoxic effects against tumor cells [3]. However, the substantial expression of TRAIL on immune cells typically requires stimuli, such as interferons [116, 117, 118, 119] or interleukins [120], which limit the apoptotic potential of endogenous TRAIL against tumor cells. Moreover, immune cells express both the membrane‐bound and soluble forms of TRAIL, yet studies have demonstrated that the membrane‐bound TRAIL exhibits higher cytotoxic activity than its soluble counterpart [51, 52]. Consequently, immobilizing recombinant sTRAIL on the membrane of immune cells is an effective strategy to enhance the antitumor efficacy of both the immune cells and the recombinant soluble TRAIL.

T cells are pivotal in the immune response against tumors, characterized by high expression levels of CD3 and CD7. However, resting T cells expressed relatively lower levels of TRAIL. Fusion of TRAIL with anti‐CD7 or anti‐CD3 antibodies facilitates its binding to the resting T cell surface, thereby converting TRAIL into a mimic membrane‐bound form [121]. Especially for anti‐CD3‐TRAIL, the anti‐CD3 component can simultaneously activate resting T cells, endowing them with intrinsic cytotoxic effector activity of T cells and leading to granzyme/perforin‐mediated tumor cell lysis. Consequently, anti‐CD3‐TRAIL concurrently enhances the tumor‐killing capabilities of resting T cells and TRAIL [121]. Similarly, neutrophils, which are more abundant than T cells in the circulatory system, also can be equipped with TRAIL. The fusion of anti‐CLL1 with TRAIL is proposed to facilitate the anchoring of TRAIL to the neutrophil surface, thereby enhancing its cytotoxicity against tumor cells and simultaneously increasing the ADCC effect of antibody drugs [122].

Although these fusion proteins are also the fusion of antibodies and TRAIL, they differ from the previously described TRAIL fusion proteins designed to enhance targeting tumor tissues. The activity of these fusion proteins is significantly enhanced only when they are co‐incubated with T cells or granulocytes.

### 4.5 Prolonging Half‐Life of TRAIL

The serum half‐life of recombinant sTRAIL is short (3–5 min in rodents, 23–31 min in nonhuman primates [123], and 0.56–1.02 h in Phase 1 trials in patients [38]), which impedes its clinical application.

The human serum albumin (HSA) and the albumin‐binding domain (ABD) are commonly utilized fusion partners to prolong the plasma half‐life of TRAIL and enhance its bioavailability, because the HSA has an exceptionally long half‐life of ~19 days [124]. The fusion of FLAG‐TNC‐TRAIL with HSA has proven to be an effective approach, as the half‐life of this fusion TRAIL in mice is increased to ~15 h, resulting in enhanced antitumor activity *in vivo* [125]. Linking HSA with TRAIL via a bifunctional PEG derivative can also improve the pharmacokinetic profiles of TRAIL, resulting in a 27‐fold increase in half‐life in mice [126]. Additionally, genetically fusing TRAIL with the ABD, a small domain that binds to albumin, has been found to significantly prolong the plasma half‐life of TRAIL by 40–50 times, leading to enhanced antitumor effects [127].

IgG molecules, particularly IgG1, IgG2, and IgG4, have exceptionally long half‐lives up to ∼18–21 days [128]. The extended half‐life of IgG is attributed to its recycling process involving Fc region binding to FcRn [124]. Thus, fusion to an Fcγ region provides therapeutic proteins with the long half‐life properties of immunoglobulins [129]. For instance, fusion of the Fcγ region of IgG1 to TRAIL significantly extends its half‐life by more than 10‐fold and enhances its antitumor efficacy *in vivo* by approximately 5‐fold. Additionally, the Fc region confers oligomeric ability to TRAIL [130]. Furthermore, the fusion of immunoglobulin‐binding domains (IgBDs) to TRAIL allows for binding to Fab fragments of IgG. IgBD‐TRAIL exhibits a significantly longer serum half‐life compared to TRAIL, with an increase of 50–60‐fold, as well as a four‐ to seven‐fold increase in tumor uptake and more than 10 times greater in vivo antitumor effect compared to TRAIL [131].

### 4.6 Overcoming Resistance of Tumor Cells to TRAIL

Cancer cells have developed diverse mechanisms to resist apoptosis triggered by TRAIL [132]. Dysregulation of TRAIL receptors, such as decreased expression of DR4 and DR5 [133], increased internalization of DR4 and DR5 [134], and overexpression of DcR1, DcR2, or OPG were observed to correlate with TRAIL resistance [9]. Upregulation of antiapoptotic proteins also plays a vital role in TRAIL resistance. Upregulation of c‐FLIP is one of the main mechanisms for cancer cells escaping from TRAIL‐induced apoptosis [135]. c‐FLIP can bind to FADD but is unable to activate caspases and thus prevents effective DISC formation [81] (Figure 1). In intrinsic apoptosis pathway, antiapoptotic proteins of the BCL‐2 family, specifically BCL‐XL, can compete with BAX for binding to tBID, whereas BCL‐2 can inhibit tBID translocating into the mitochondrial outer membrane (Figure 1). Overexpression of BCL‐XL or BCL‐2 is associated with tumor cells' resistance to TRAIL [136]. IAP family proteins regulate both the extrinsic and intrinsic pathways (Figure 1). XIAP has been shown to interact with caspases 3, 7, and 9 and inhibits their activities, whereas cellular inhibitor of apoptosis protein (cIAP)1 and cIAP2 polyubiquitinates caspases 3 and 7, leading to their degradation. XIAP, cIAP1, and cIAP2 are frequently overexpressed in a number of cancers and revolved in cell death resistance [137].

It was found that decreased levels of phosphorylated Akt (p‐Akt) led to reduced expression of c‐FLIP, thereby alleviating TRAIL resistance in cancer cells. Iron superoxide dismutase (FeSOD) was shown to decrease p‐Akt levels. Consequently, the fusion protein sTRAIL:FeSOD resulted in the downregulation of both p‐Akt and c‐FLIP, thereby promoting apoptosis in TRAIL‐resistant cells [138]. AD‐O57.4 and AD‐O57.5 are fusion proteins composed of TRAIL and the BH3 domain of BID connected by distinct linkers. Both constructs have demonstrated the ability to induce apoptosis in cell lines that were initially resistant to TRAIL [139]. The IAPs impede the activation of downstream caspases, and the mitochondrial protein SMAC/Diablo can eliminate the inhibitory effects of XIAP on caspase activation [140]. A fusion protein, AD‐O53.2, was designed to combine sTRAIL with a peptide derived from SMAC/Diablo protein (AVPIAQKP) in order to facilitate the delivery of the SMAC/Diablo peptide to the cytoplasm for the purpose of inhibiting XIAP and overcoming TRAIL resistance [141]. Similarly, in another study, the 10 aa in the N‐terminal of the sTRAIL was replaced with RRRRRR (a cell penetrating peptide polyarginine) and AVPI (the N‐terminal and binding site of SMAC). The resulted protein, named R6ST, exhibited improved affinity to DR4 and DR5, and significantly decreased XIAP intracellular concentration in PANC‐1 cells compared with sTRAIL [142]. The fusion protein AD‐O56.9 also demonstrated significant tumor regression in mouse xenograft models of TRAIL‐resistant tumors. It consists of sTRAILand the cationic, alpha‐helical (KLAKLAK) 2 antimicrobial peptide, which is known for its potent ability to induce apoptosis by disrupting the mitochondrial membrane [143]. Another fusion protein SAC‐TRAIL consists of TRAIL and SAC protein. SAC is the core domain (aa137–195) as well as the effector domain of prostate apoptosis response‐4, which was identified as essential for TRAIL‐induced apoptosis [144]. The two functional components of SAC‐TRAIL demonstrated synergistic effects, resulting in enhanced antitumor efficacy compared to TRAIL alone [145].

### 4.7 Other TRAILFusions

There are also constructs of TRAIL fusions that demonstrated higher antitumor efficacy than native TRAIL without accurate mechanism, for example, the antibacterial peptide CM4 fused with TRAIL induced more potent apoptosis than that of TRAIL [146]. Another chimeric protein Annexin V‐TRAIL triggered robust apoptosis in A549 cells and demonstrated significant inhibition of tumor growth in A549 xenografts, which is resistant to TRAIL [147]. Additionally, TRAIL‐Mu3, a mutant form of wild‐type TRAIL, is not a typical TRAIL fusion protein. The 114–121 amino acid sequence “VRERGPQR” of TRAIL was mutated into a membrane‐penetrating peptide‐like amino acid sequence “RRRRRRRR.” This modification resulted in increased affinity for the cell membrane and enhanced proapoptotic potential [148].

## 5 Conclusion and Perspective

TRAIL is an endogenous apoptosis‐inducing ligand that specifically kills cancer cells with little harm to normal cells, which makes it a promising molecule for cancer treatment. However, clinical trials of sTRAIL showed limited efficacy, due to its relatively short half‐life, decreased protein stability, insufficient tumor accumulation, and resistance exhibited by cancer cells. An increasing number of TRAIL fusion proteins have been constructed over the past few years, and potent approaches have been successfully employed to create multifunctional TRAIL. Notably, some of them have shown sufficient promise to advance into clinical trials [78, 79, 149, 150]. Nevertheless, significant challenges remain in TRAIL fusion protein development.

Hepatotoxicity has been a major limitation for the clinical use of TNF superfamily members [151]. Certain versions of tagged TRAIL, such as His‐TRAIL [152], FLAG‐TRAIL, or LZ TRAIL [153], have been reported to induce apoptosis in human hepatocytes. This hepatotoxicity has been attributed to the self‐aggregation of tagged TRAIL due to the lack of Zn^2+^ ions and reduced solubility [152], or to the artificial receptor cross‐linking by the addition of FLAG antibody [153], which induces a stronger signal through the DRs in hepatocytes. Consistent with these observations, in our previous study [72], we observed fatal hepatotoxicity in BALB/c nude mice administered a high dose (40 mg/kg) of His‐TRAIL. In addition, in a nonclinical toxicology study, recombinant human TRAIL (without any tag) also demonstrated hepatotoxicity in cynomolgus monkeys. Researchers determined that cross‐species antitherapeutic antibodies in cynomolgus monkeys crosslinked recombinant human TRAIL, inducing the aggregation of hepatocyte DRs and amplifying downstream signaling leading to apoptosis [154]. Similarly, in a Phase 1 study of TAS266, a tetravalent agonistic nanobody targeting the DR5 receptor, three patients developed TAS266‐related hepatotoxicity. The possible cause is suspected to be related to the multivalence of the molecule, which efficiently enhances DR5 clustering [155].

Therefore, minimizing hepatoxicity is crucial in the TRAIL fusion protein design, particularly ensuring that potency and valency enhancement should not be pursued at the expense of safety. One approach to attenuate hepatotoxicity is to enhance the tumor‐targeting specificity of TRAIL fusion proteins, as detailed in the text. Alternatively, optimized delivery systems can be employed. For example, therapeutic engineered neural stem cells were developed to be implanted in the resection cavity of glioblastoma in mice, offering a continuous and concentrated local delivery of sTRAIL, thus reducing its nonselective targeting [156]. In summary, it is advisable to concurrently evaluate the hepatotoxicity of TRAIL fusion proteins alongside their antitumor activity.

Notably, the existence of resistance of tumor cells against TRAIL fusion proteins should be considered during the design and development of TRAIL fusion proteins. Combination with other drugs is a common strategy to overcome resistance. For example, the combination of cyclopamine and CPT exhibited synergistic effects on inhibition, proliferation, and inducing apoptosis in myeloma cells [157]. For ABBV‐621, researchers have demonstrated that the inherent resistance of cancer cells to ABBV‐621 treatment can be overcome in combination with chemotherapeutics or with selective inhibitors of BCL‐XL, A‐1331852 [69]. Furthermore, an ongoing Phase 1b clinical trial (NCT04570631) is evaluating the efficacy of ABBV‐621 in combination with bortezomib and dexamethasone in patients with relapsed or refractory multiple myeloma [79]. It is also necessary to identify appropriate biomarkers for patients to determine their suitability for TRAIL treatment and to select the most effective sensitizers for TRAIL fusion proteins to achieve better therapeutic efficacy. In addition to combination with TRAL sensitizers, meticulously designed delivery systems can also enhance tumor cell sensitivity to TRAIL. For example, a nanocage was designed to deliver TRAIL in its native‐like trimeric structure, along with doxorubicin, which re‐sensitizes TRAIL‐resistant tumor cells [158]. Another nanoplatform, named CPT MV, is a novel reactive oxygen species (ROS)‐dependent TRAIL‐sensitizing nanoplatform, featuring a Ce6‐PLGA core and a TRAIL‐modified cell membrane shell. Upon laser irradiation, it generates ROS in targeted cancer cells, improving DR5 expression, triggering cytochrome c release from mitochondria, and strengthening TRAIL‐induced apoptosis [159]. Furthermore, considering that DcRs can competitively block TRAIL's interaction with DR4 and DR5, potentially conferring resistance to TRAIL in tumor cells [160, 161], and that DcR2 may induce non‐apoptotic signaling pathways [22], using DR4‐specific [162] or DR5‐specific [163, 164] TRAIL variants as the main body for fusion could represent a viable option [93].

We note that while a small portion of fusion protein designs utilize computer‐aided design, the majority still rely on empirical screening approaches with high uncertainty. Unexpected outcomes, including improper folding, reduced biological activity, and unforeseen toxicity, frequently arise, highlighting our limited understanding of the structure–function relationships in these fusion proteins [165]. Nowadays, computational methods have revolutionized protein engineering, allowing a diverse set of biocomputing capabilities such as prediction of protein three‐dimensional models, characterization of protein–protein interactions, and the molecular dynamics simulations [166]. We believe that prevalidation of fusion protein conformational state and overall quality through biocomputing methods will make the design of TRAIL fusion proteins more efficient and rational.

Overall, we are inclined to believe that an optimal strategy for engineering TRAIL fusion proteins should ideally address all the three issues: pharmacokinetics, pharmacodynamics, and resistance. Among these, the most critical goal should be increasing the oligomerization of recombinant TRAIL without the formation of aggregates. Other desirable attributes might encompass improved tumor targeting, the ability to overcome resistance, and prolonged half‐life. It is imperative to emphasize that vigilance on hepatotoxicity should accompany the entire process, from the initial design to the validation of antitumor activity.

Notwithstanding a number of challenges in the way, TRAIL remains one of the most promising approaches for cancer treatment. Rational design of fusion proteins, or further development based on this approach, could potentially provide opportunities for the clinical use of TRAIL‐based therapeutics.

## Author Contributions

**Yan Wang:** conceptualization (lead), data curation (equal), funding acquisition (lead), visualization (equal), writing – original draft (lead), writing – review and editing (lead). **Xin Qian:** data curation (equal), visualization (equal), writing – original draft (equal), writing – review and editing (equal). **Yubo Wang:** data curation (equal). **Caiyuan Yu:** data curation (equal), writing – review and editing (supporting). **Li Feng:** data curation (equal), writing – review and editing (supporting). **Xiaoyan Zheng:** data curation (equal). **Yaya Wang:** data curation (equal). **Qiuhong Gong:** conceptualization (supporting), supervision (lead), writing – review and editing (supporting).

## Conflicts of Interest

The authors declare no conflicts of interest.

## Data Availability

The authors have nothing to report.
